# The diagnostic yield of whole exome sequencing as a first approach in consanguineous Omani renal ciliopathy syndrome patients

**DOI:** 10.12688/f1000research.40338.1

**Published:** 2021-03-12

**Authors:** Intisar Al Alawi, Mohammed Al Riyami, Miguel Barroso-Gil, Laura Powell, Eric Olinger, Issa Al Salmi, John A. Sayer

**Affiliations:** 1 Translational and Clinical Research Institute, Newcastle University, Newcastle upon Tyne, Tyne and Wear, NE13BZ, UK; 2 National Genetic Center, Ministry of Health, Muscat, Oman; 3 Renal Medicine Department, Royal Hospital, Ministry of Health, Muscat, Oman; 4 Royal Hospital, Ministry of Health, Muscat, Oman; 5 Renal Services, Oman Medical Speciality Board, Newcastle upon Tyne, Tyne and Wear, NE77DN, Oman; 6 Newcastle Biomedical Research Centre, NIHR, Newcastle upon Tyne, Tyne and Wear, NE45PL, UK

**Keywords:** renal ciliopathy, cystic kidney disease, Oman, whole exomes sequencing

## Abstract

**Background:** Whole exome sequencing (WES) is becoming part of routine clinical and diagnostic practice. In the investigation of inherited cystic kidney disease and renal ciliopathy syndromes, WES has been extensively applied in research studies as well as for diagnostic utility to detect various novel genes and variants. The yield of WES critically depends on the characteristics of the patient population.

**Methods:** In this study, we selected 8 unrelated Omani children, presenting with renal ciliopathy syndromes with a positive family history and originating from consanguineous families. We performed WES in affected children to determine the genetic cause of disease and to test the yield of this approach, coupled with homozygosity mapping, in this highly selected population.

DNA library construction and WES was carried out using SureSelect Human All Exon V6 Enrichment Kit and Illumina HiSeq platform. For variants filtering and annotation Qiagen Variant Ingenuity tool was used. Nexus copy number software from BioDiscovery was used for evaluation of copy number variants and whole gene deletions. Patient and parental DNA was used to confirm mutations and the segregation of alleles using Sanger sequencing.

**Results:** Genetic analysis identified 4 potential causative homozygous variants each confirmed by Sanger sequencing in 4 clinically relevant ciliopathy syndrome genes, ( *TMEM231*, *TMEM138*, *WDR19* and *BBS9*), leading to an overall diagnostic yield of 50%.

**Conclusions:** WES coupled with homozygosity mapping provided a diagnostic yield of 50% in this selected population. This genetic approach needs to be embedded into clinical practise to allow confirmation of clinical diagnosis, to inform genetic screening as well as family planning decisions. Half of the patients remain without diagnosis highlighting the technical and interpretational hurdles that need to be overcome in the future.

## Introduction

There are over 750 million people worldwide affected with chronic kidney disease (CKD), a disease burden that is much higher than those living with diabetes, cancer or even AIDS/HIV ^1^. Inherited kidney diseases and renal ciliopathy syndromes are one of the major contributors to CKD burden, where up to 10% of adults and over 70% of children reaching end stage kidney disease (ESKD) are expected to harbour genetic causes ^2^. However, studying such rare diseases has considerable challenges mainly due to the small size of patient cohorts negatively affecting progress of treatments and commercial feasibility. Collaborative research and progress of new technologies and methodologies are strategic to overcoming these challenges.

WES is becoming part of routine clinical and diagnostic practice ^2^. Focusing only on protein-coding regions through WES decreases the sequencing costs and produces manageable genetic data for interpretation, which enhances its extensive usage in diagnosis leading to the discovery of previously unrecognized renal disease genes and disorders ^2, 3^. In the case of heterogeneous renal ciliopathies, WES has been extensively applied in research studies as well as for diagnostic utility to detect various novel genes and variants ^4, 5^. In this study, WES was used to determine the genetic causes of cystic kidney disease and renal ciliopathy syndromes in a group of 8 unrelated Omani children from consanguineous families. As this study shows, the focus of nephrogenetics in Oman is primarily to establish an accurate genetic diagnosis to explain clinical phenotypes using the significantly improved diagnostic power of genomic technologies.

## Methods

### Ethical approvals and patients’ inclusion and clinical evaluation

This study was approved by the North East-Newcastle & North Tyneside 1 Research Ethics Committee (18/NE/350).

Patients were identified and recruited from paediatric referrals for investigation of inherited kidney disease to the nephrology services within the Ministry of Health Hospital, Muscat, Oman between 2015 and 2018. Whole blood (1.5-2.5 ml in EDTA) samples were collected specifically for this study and used for extraction of genomic DNA. DNA samples from affected and other family members were given an anonymised sample number. All patients had clinical features strongly suggestive of an inherited renal ciliopathy. Written and informed consent was obtained from the parents / guardians of each patient, and any family members (including parents and siblings) involved in this study.

Clinical information relating to patient presentation, phenotype and family pedigree structure, with an emphasis on familial kidney disease was obtained, following informed consent for access to the medical records. Family pedigrees were drawn using Invitae ^©^ online tool ( https://familyhistory.invitae.com).

### DNA isolation, library preparation and exome sequencing

gDNA was isolated from whole blood of patients and the available family members using Hamilton’s Microlab® STAR™, according to the manufacturer protocol. DNA extraction was performed in the National Genetic Centre in Oman. DNA library construction and WES were outsourced to EuroFins GATC Biotech (Germany) or Novogene Co., Ltd (China). SureSelect Human All Exon V6 Enrichment Kit (Agilent Technologies, CA, USA) and Illumina HiSeq platform (Illumina, San Diego, CA, USA) were used. Analyses of raw data (FASTQ format) were performed including sequence reads mapping to the human reference genome hg19 using BWA (Li and Durbin, 2009), removal of PCR duplicates using Picard ( http://broadinstitute.github.io/picard/), alignment refinement using GATK, coverage analysis and SNP and indel calling using GATK’s Haplotype Caller (McKenna *et al.*, 2010).

### Variant and CNV detection and annotation

SNP and indel VCF files were investigated using Qiagen Variant Ingenuity tool for variants filtration and annotation. Nexus copy number software from BioDiscovery (9.0) was used for CNVs analysis and visualization. To detect regions of homozygosity, WES genotype data were used to create homozygosity mapping using the online homozygosity mapper tool ( http://www.homozygositymapper.org/).

### Variant validation by Sanger sequencing

Sanger sequencing was utilized to confirm suspected disease-causing variants and their segregation if DNA samples from parents and other family members were available. Primer3 was utilized to design primer sequences ( http://primer3.ut.ee/) (Extended Data Table 1 ^6^). PCR amplification was performed using *Taq* PCR master mix (Qiagen) kit, as per the manufacturer instructions. Sanger sequencing was outsourced to EuroFins GATC Biotech (Germany). The obtained sequences were assembled and aligned compared to a reference sequence using the SequencePilot 4.2.2 software (JSI Medical Systems GmbH).

## Results

### Patient characteristics

WES was carried out for 8 unrelated paediatric patients with an age range of 3 months to 6 years of age (5 female, 3 male) with a clinical suspicion of a renal ciliopathy syndrome and known consanguinity as demonstrated by pedigree diagrams (Extended data Figure 1 ^6^). This was a diagnostic-naïve population without prior genetic analysis. Patients had a variety of clinical features, renal and extra-renal, with 5 probands reaching ESKD within 5 years of life ( Table 1). Seven out of 8 had a positive family history of kidney disease and 6 had extra-renal manifestations typical of ciliopathy syndromes which included Senior-Løken syndrome, Joubert syndrome, Meckel syndrome and Bardet-Biedl syndrome ( Table 1). 

**Table 1.** Clinical characteristics of Omani patients.

Patient ID	Gender	Age at referral	Clinical features	Additional clinical features	CKD stage	Family history of kidney disease	Parental consanguinity
M43	F	3 y	Nephronophthisis	DD, right hip dysplasia, failure to thrive.	5 (ESKD at 3 y)	Yes	Yes
M44	F	2 y	Cystic kidney disease	Hypertension, liver fibrosis	5 (ESKD at 2 y)	Yes	Yes
M46	F	3 m	Meckel syndrome with cystic kidneys	Dysmorphic features, occipital encephalocele, polydactyly, diaphragmatic hernia	5 (ESRD at 1 y)	Yes	Yes
M47	F	5 y	Cystic kidney disease	Retinitis pigmentosa, conductive hearing loss	5 (ESKD at 5 y)	Yes	Yes
M48	M	3 y	Joubert syndrome with cystic kidneys	DD, hypotonia, poor visual acuity, brain MRI showed molar tooth malformation	1	Yes	Yes
P3	M	3 y	Cystic kidney disease		1	No	Yes
P18	M	6 y	Nephronophthisis	Hypertension, DD and retinal dystrophy.	5 (ESKD at 5 y)	Yes	Yes
N36	F	1 y	Cystic kidney disease	Post-axial polydactyly	1	Yes	Yes

CKD, chronic kidney disease; DD, developmental delay; ESKD, end stage kidney disease; F, female; M, male; m, month; y, year.

### Exome sequencing data

Quality control of WES revealed that >99% of the reads were properly mapped to the reference genome. The details of the depth, coverage and target sequences covered are summarized in Extended data Table 2 ^6^. The average coverage depth was 145.9. Comparable coverage of target coding regions was achieved among the 8 cases with an average of 96.4% of the exome being covered at least 20-fold (Extended data Table 2 ^6^). Homozygosity mapping of all patients confirmed large regions of homozygosity, typical of known parental consanguinity (Extended data Figure 2 ^6^).

### Molecular genetic findings

**Figure 1.**
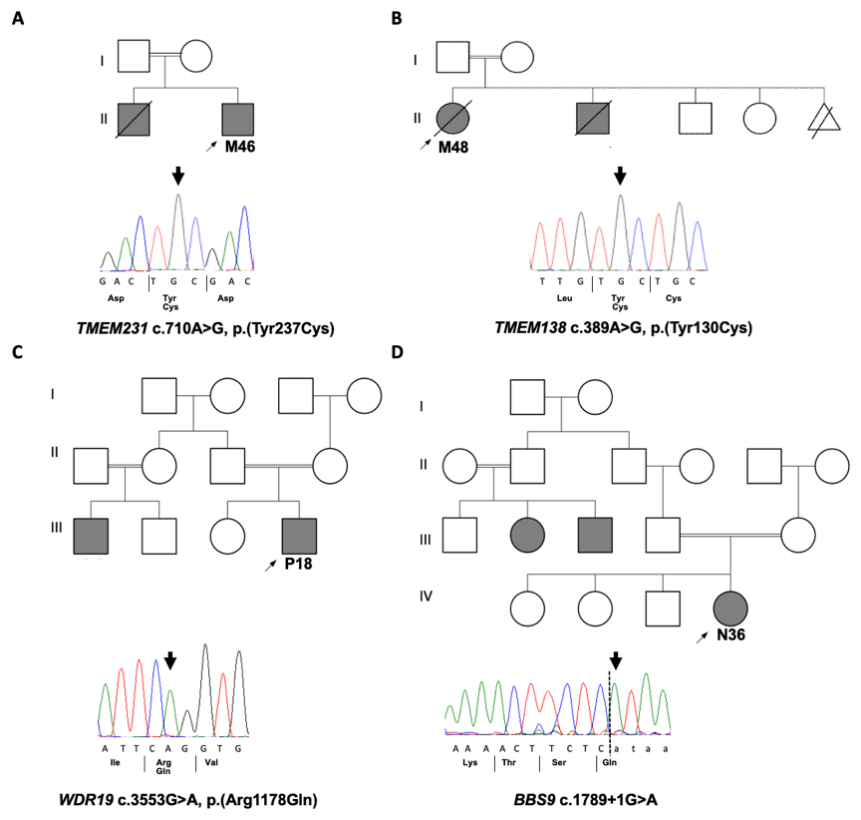
Family structures and Sanger sequencing in solved renal ciliopathy cases. Pedigrees of solved families with Sanger sequecing chromatograms confirming the disease causative variants that were identified by WES in four families. **A**. M46 with homozygous missense variant in *TMEM231*. **B**. M48 with homozygous missense variant in *TMEM138*. **C**. P18 with homozygous missense variant in *WDR19* **D**. N36 with homozygous splice site variant in *BBS9*.

**Figure 2.**
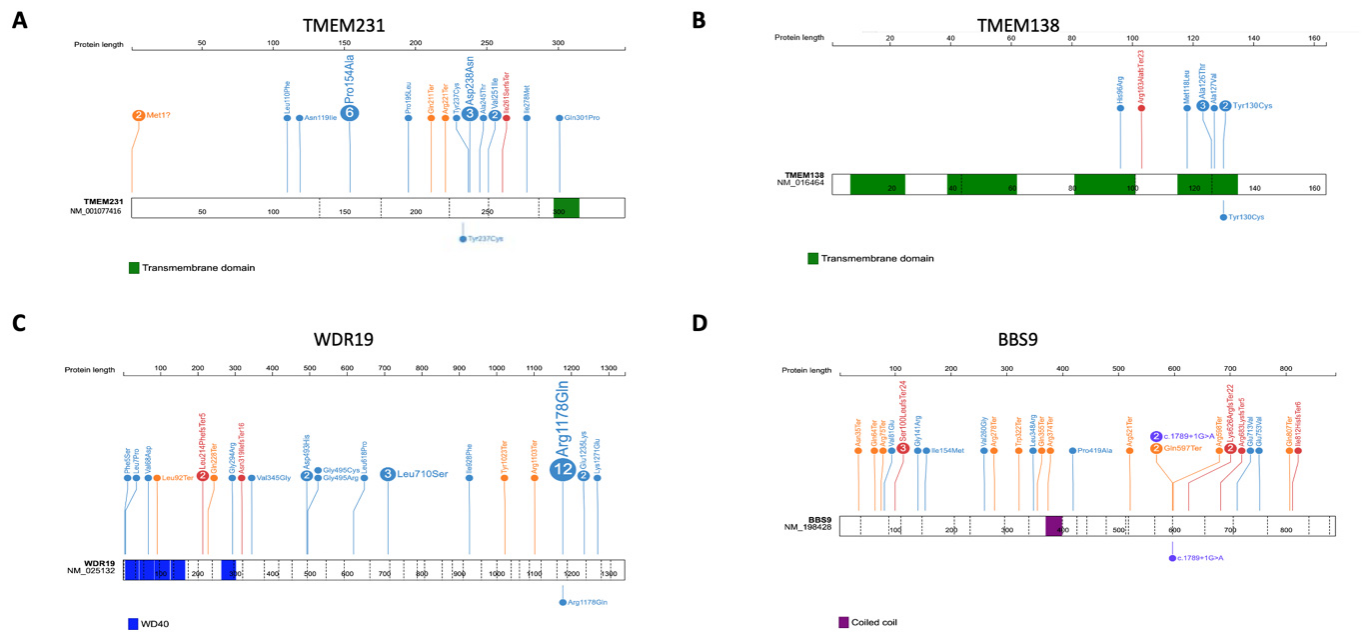
Distribution of mutations in *TMEM231*, *TMEM138*, *WDR19* and *BBS9*. Positions and the predicted protein alterations are shown for **A**. *TMEM231* **B**. *TMEM138* **C**. *WDR19* **D**. *BBS9*. Exon structure is marked by a dashed line. Protein domains are shown in colored bars. Known mutations are shown above the gene/protein structure with the number showing frequency (if >1) of probands reported for each mutation. Mutations identified in this study are shown below the gene/protein structure.

A molecular genetic diagnosis was obtained in 4 out of the 8 patients ( Figure 1), leading to an overall diagnostic yield of 50% ( Table 2). Four different homozygous single nucleotide variants (SNVs) were detected in 4 known ciliopathy genes ( *TMEM231*, *TMEM138*, *WDR19* and *BBS9*) and were confirmed by Sanger sequencing ( Figure 1). Three of the mutations were missense mutations affecting highly conserved amino acids (Extended Data Figure 3 ^6^) whilst the fourth was a splice-site mutation ( Figure 2). All tested samples were examined for mutations in ACMG actionable genes but none were identified.

**Table 2.** Molecular Genetic Findings in four Omani children with renal ciliopathy syndromes.

Family - individual	Gene name	Nucleotide change	Amino acid change	Zygosity	Amino acid conser.	ACMG Classification	dbSNP ID	MAF	CADD score	SIFT Pred	PolyPhen-2 Pred	MutationTaster	Reference
M46	*TMEM231*	c.710A>G	p.Y237C	Hom	*C.elegans*	Uncertain significance	NA	Not found	22.7	Damaging	Possibly Damaging	Disease causing	N/A
M48	*TMEM138*	c.389A>G	p.Y130C	Hom	*D.rerio*	Likely pathogenic	rs387907135	3.98×10 ^-6^ (gnomAD)	25.7	Deleterious	Probably damaging	Disease causing	Lee *et al.* (2012) ^7^
P18	*WDR19*	c.3553G>A	p.R1178Q	Hom	*C.elegans*	Likely pathogenic	rs79436363	6.35×10 ^-5^ (gnomAD)	24.6	Tolerated	Probably Damaging	Disease causing	Halbritter *et al.* (2013) ^8^
N36	*BBS9*	c.1789+1G>A	Splice donor site loss	Hom	N/A	Pathogenic	rs201938124	7.96×10 ^-6^ (gnomAD)	25	N/A	N/A	Disease causing	Nishimura *et al.* (2005) ^13^

Reference sequence IDs: *TMEM231*: NM_001077416; *TMEM138*: NM_016464; *WDR19*: NM_025132; *BBS9*: NM_198428 Abbreviations: CADD score, combined annotation dependant depletion; conser, conservation; gnomAD, Genome Aggregation Database; Hom, homozygous; N/A, not available

The identified causative variant in M46 was novel (c.710A>G; p.Y237C in *TMEM231*) and has not been previously reported in any databases. This homozygous missense change is found in a large region of homozygosity on Chromosome 16 (Extended data Figure 2 ^6^) and is predicted by Sorting Intolerant from Tolerant (SIFT) to be damaging, PolyPhen-2 to be possibly damaging and MutationTaster to be disease causing. The Y237 amino acid in TMEM231 is conserved to *Caenorhabditis elegans* (Extended data Figure 3 ^6^). Mutations in *TMEM231* are known to cause both Joubert syndrome and Meckel syndrome (Extended Data Table 3 ^6^), and the clinical phenotype of patient M48, which included encephalocele, polydactyly and polycystic kidney disease and early onset ESKD, is consistent with a Meckel-like ciliopathy syndrome.

The identified causative variant in M48 was a known allele (c.389A>G; p.Y130C in *TMEM138*) and has been previously reported in a child with Joubert syndrome and a cerebello-retinal-renal phenotype ^7^. This homozygous missense change is found in a narrow region of homozygosity on Chromosome 11 (Extended data Figure 2 ^6^) and is predicted by SIFT to be deleterious, PolyPhen-2 to be probably damaging and MutationTaster to be disease causing. The Y130 amino acid in TMEM138 is conserved to *Danio rerio* (Extended data Figure 3 ^6^). Mutations in *TMEM138* are known to cause Joubert syndrome (Extended data Table 4 ^6^), and the clinical phenotype of patient M48, which included molar tooth sign, visual loss and cystic kidney disease, is consistent with a Joubert syndrome ciliopathy.

The identified causative variant in P18 was a known allele (c.3553G>A; p.R1178Q) in *WDR19* and has been previously reported in cases of nephronophthisis (NPHP)-related ciliopathies with retinal and liver involvement ^8– 10^, Senior-Løken syndrome ^11^ and more complex ciliopathies ^12^. This homozygous missense change is found in a large region of homozygosity on Chromosome 4 (Extended data Figure 2 ^6^) and segregation of the pathogenic causative allele in *WDR19* with P18’s family members was confirmed. The missense allele is predicted by SIFT to be tolerated, PolyPhen-2 to be probably damaging and MutationTaster to be disease causing. The R1178 amino acid in WDR19 is conserved to *C.elegans* (Extended data Figure 3 ^6^). Mutations in *WDR19* are associated with a wide spectrum of ciliopathies (Extended data Table 5 ^6^), and the clinical phenotype of patient P18, which included NPHP and early onset ESKD and retinal dystrophy is consistent with a Senior-Løken syndrome.

The identified causative variant in N36 was a known splice-site allele (c.1789+1G>A in *BBS9*) and has been previously reported in patients with Bardet-Biedl syndrome (BBS) ^13, 14^. This homozygous missense change is found in a region of homozygosity on Chromosome 7 (Extended data Figure 2 ^6^) and is predicted to cause loss of splice donor site. Mutations in *BBS9* are known to cause BBS (Extended data Table 6 ^6^), and the clinical phenotype of patient N36, which included features of BBS including post-axial polydactyly affecting all limbs and cystic kidney disease is consistent with a BBS ciliopathy.

## Discussion

In paediatric populations, CKD is a major contributor to health-care burden leading to severe morbidity and mortality. At least 17% of those with ESKD are considered as CKD with unknown aetiology, where the primary kidney disease is not clear ^15^. In addition, the primary clinical diagnosis of CKD patients is often inaccurate ^15^. Thus, in the developing era of precision medicine, WES is used as an essential tool that provides novel diagnostic perspectives for the detection of the causes of CKD. Knowledge of genetic causes has valuable clinical implications in therapeutic intervention, improving prognosis, guide family counselling or managing settings of kidney transplantation ^16^. Despite being rare, inherited kidney diseases represent one of the most common causes of CKD and ESKD, accounting for up to 10% of adults and almost all children commencing renal replacement therapy ^17^. The possibility of monogenic causes in those with unknown aetiology of CKD or with atypical clinical presentation is assumed to be high ^15^. At least 500 different genetic causes have been associated with childhood CKD ^18^.

In this pilot study, we examined the utility of WES in the diagnosis of 8 different Omani children with childhood onset CKD related to cystic kidney disease and a suspected inherited renal ciliopathy. A conclusive genetic diagnosis was achieved in half of the cases. Positive WES findings allow a precise molecular diagnosis and targeted clinical management as well as informing family planning and facilitating proper genetic counselling. In four of the children (M46, M48, P18 and N36) the molecular genetic findings confirmed the suspected clinical diagnosis. The identification of a molecular genetic diagnosis in all these families can provide accurate genetic advice about the parent’s reproductive choices and the possibility of preimplantation genetic diagnosis (PGD) or early genetic testing of a foetus in future pregnancies.

A wide range of genetic studies have been performed in childhood CKD populations and different diagnostic yields were achieved due to differences in the inclusion criteria or patients and the study design. In a study of families with inherited kidney disease, Mallett *, et al.* ^19^ reported a diagnostic yield of 46%, reflecting the significant ability of WES in underlying the potential genetic causes of most renal phenotypes. In another recent study ^2^, Groopman *et al*. reported higher diagnostic yield in patients with congenital and cystic kidney disease (23.9%). Furthermore, regardless of the primary kidney diagnosis, higher diagnostic yield was associated with a positive family history of CKD, history of parental consanguinity and presentations of extra-renal features ^2, 5^. Thus using a combination of homozygosity mapping along with WES genotype data is always recommended as a powerful approach for consanguineous families to identify rare genetic causes ^20^.

Although WES provides massive amounts of genetic data, 4 patients remained unsolved in this study. Interpretation of many novel and extremely rare variants is still limited by the incomplete knowledge of the total human protein-coding genes as well as the incorrect annotation of variants pathogenicity and incorrect association of genes with the disease in the literature. At present, up to 70% of protein-coding genes have no recognized human disease phenotype ^21^. False gene-disease associations are present in the literature ^22, 23^ and clinically valuable databases of variants pathogenicity, such as Human Gene Mutation Database (HGMD ^®^), comprise various errors causing benign variants being falsely selected out of the data and allocated as plausible diagnosis ^24^. This situation is predicted to improve as further genomes are sequenced, including large data collections containing populations of both healthy individuals and patients with rare diseases. In addition, studying more families with similar clinical phenotypes from the same population may facilitate linking novel undiscovered genes to the disease phenotype in those unsolved patients.

In this study, WES confirmed the clinical diagnosis in 4 children. In a similar study of large consanguineous or familial cohort ( *n* = 79) of children clinically diagnosed with NPHP, genetic diagnostic yield of 63% was reported, of which the clinical diagnosis was confirmed in 64% and changed to different molecular diagnosis in the remaining 36% ^10^.

This study has some limitations, including small sample size that does not give a generalized image of broader childhood renal ciliopathy in the population from Oman. However, an enhanced assessment of the utility of WES in the clinical diagnostic practice of these disorders may be given through systematic WES analysis of a larger, unselected cohort. Moreover, the diagnostic gap in this study may be caused by the common technical limitation of WES, including the missed detection of structural variant breakpoints, sequencing difficulties for regions with repetitive elements or guanine-cytosine (GC)-rich regions, and limited discrimination between highly homologous genomic regions with pseudogenes. These limitations are attributed to the short-read lengths that are utilized to generate high genomic coverage and depth ^25^. These limitations are assumed to be resolved through using long-read sequencing platforms that compromise these technical challenges and improve the detection of genetic variants ^25^. Thus, the emerging future of long-read sequencing based whole genome sequencing (WGS) could enhance the diagnostic yield of patients with inherited renal ciliopathies and provide more conclusive primary kidney disease diagnosis. This can be supported by recent reports of WGS obtaining higher molecular diagnostic yield compared with WES, where 20–40% of those unsolved by WES were genetically conclusive by WGS ^26^.

Recent advancements in medical genetics through the use of massively parallel sequencing have not only advanced the discovery of novel causative variants, genes and phenotypes, but also contributed to the re-classification of diseases and phenotypes into novel gene-based ontologies ^27^. However, all types of next generation sequencing (NGS)-based testing (Target panel, WES and WGS) have some shared limitations, including the inability to obtain enough coverage of genomic regions with highly repetitive GC-content sequence, such as that in *MUC1* gene. In his study of six unrelated families with medullary cystic kidney disease type 1 (MCKD1) ^28^, Kirby *et al.* highlighted the challenges of these technologies in detecting the causative monogenic causes of some Mendelian disorders, such as MCKD1, where only long-range polymerase chain reaction and molecular cloning successfully performed the task. Moreover, in many patients with acquired diseases, NGS testing is of limited importance and transformation of genetic results into clinical setup may be challenging ^27^. In the field of kidney disease, the majority of genetic testing studies are narrowed to a research setting, thus until now the knowledge of its diagnostic efficacy in clinical practice is still limited ^15^. In addition, managing the medical ethics raised by these technologies, including uncertain variants and incidental findings, and balancing the social concerns is still challenging ^29^.

## Conclusion

WES of patients with different inherited cystic kidney diseases and renal ciliopathies shows promise as a diagnostic tool, especially in well selected patients with a high coefficient of inbreeding and/or with a syndromic presentation. It has the potential to resolve those cases with clear suspicion of renal ciliopathies, as well as those with uncertain aetiology causing CKD. The fact that ~50% of patients remain without genetic diagnosis after WES highlights the need for improved sequencing techniques and interpretation tools, driven by constantly evolving knowledge regarding the genetic architecture of diseases. The clinical impacts of positive WES results on therapeutic choice, genetic counselling and guidance of kidney transplant are critical. Indeed, professional genetic counselling on the prospective effects of a positive test result is crucial, bearing also in mind the possibility of incidental findings. Although further studies from the Omani population are required, we predict an expanding impact of NGS-based diagnosis, both gene panels and WES in clinical practice in the very near future.

## Data availability

### Underlying data

Figshare: The diagnostic yield of whole exome sequencing as a first approach in consanguineous Omani renal ciliopathy syndrome patients, https://doi.org/10.6084/m9.figshare.13696750.v1 ^30^.

This project contains the following underlying data:

- M46.snps.vcf
- M46.indels.vcf
- M48.snps.vcf
- M48.indels.vcf
- JAS_P18.GATK.snp.vcf
- JAS_P18.GATK.indel.vcf
- JAS_N36.GATK.snp.vcf
- JAS_N36.GATK.indel.vcf

### Extended data

Figshare: The diagnostic yield of whole exome sequencing as a first approach in consanguineous Omani renal ciliopathy syndrome patients, https://doi.org/10.6084/m9.figshare.c.5287753.v1 ^6^.

This project contains the following extended data:

- Extended data Table 1. Forward and reverse primer sequences used for WES variants verification ( https://doi.org/10.6084/m9.figshare.13675201)
- Extended data Table 2. Whole exome sequence alignment and coverage profile by sample ( https://doi.org/10.6084/m9.figshare.13675222)
- Extended Data Table 3. TMEM231 alleles ( https://doi.org/10.6084/m9.figshare.13675471)
- Extended data Table 4. TMEM138 alleles ( https://doi.org/10.6084/m9.figshare.13675504.v1)
- Extended data Table 5. WDR19 alleles ( https://doi.org/10.6084/m9.figshare.13675540)
- Extended data Table 6. BBS9 alleles ( https://doi.org/10.6084/m9.figshare.13675546)
- Extended data Figure 1. Pedigree diagrams ( https://doi.org/10.6084/m9.figshare.13675552.v1)
- Extended data Figure 2. Homozygosity mapping ( https://doi.org/10.6084/m9.figshare.13675558.v1)
- Extended data Figure 3. Clustal alignments of amino acids associated with identified missense mutations ( https://doi.org/10.6084/m9.figshare.13675561.v1)

Data are available under the terms of the Creative Commons Attribution 4.0 International license (CC-BY 4.0).
