# Supplementary material for: The diagnostic yield of whole exome sequencing as a first approach in consanguineous Omani renal ciliopathy syndrome patients
Source: F1000Res. 2021 Jul 7;10:207. Originally published 2021 Mar 12. [Version 2] doi: 10.12688/f1000research.40338.2 (PMC8290205; doi:10.12688/f1000research.40338.2)
Supplement: Peer review history

# Peer review: Reviewer response for version 2

**DOI:** 10.5256/f1000research.58306.r89233

**Published:** 2021-07-19

**Authors:** Paraskevi Goggolidou

**Affiliations:** 1 Department of Biomedical Science and Physiology, Faculty of Science and Engineering, University of Wolverhampton, Wolverhampton, UK

Having looked at the revised manuscript, I am happy to accept it.

Is the work clearly and accurately presented and does it cite the current literature?

Yes

If applicable, is the statistical analysis and its interpretation appropriate?

Not applicable

Are all the source data underlying the results available to ensure full reproducibility?

Yes

Is the study design appropriate and is the work technically sound?

Yes

Are the conclusions drawn adequately supported by the results?

Partly

Are sufficient details of methods and analysis provided to allow replication by others?

Yes

Reviewer Expertise:

NA

I confirm that I have read this submission and believe that I have an appropriate level of expertise to confirm that it is of an acceptable scientific standard.

# Peer review: Reviewer response for version 2

**DOI:** 10.5256/f1000research.58306.r89232

**Published:** 2021-07-12

**Authors:** Enza Maria Valente

**Affiliations:** 1 Neurogenetics Research Center, IRCCS Mondino Foundation, Pavia, Italy; 2 Department of Molecular Medicine, University of Pavia, Pavia, Italy

I am satisfied with the changes that the authors made to the article. A significant section on negative results has been added which now makes the manuscript more complete and informative.

Is the work clearly and accurately presented and does it cite the current literature?

Yes

If applicable, is the statistical analysis and its interpretation appropriate?

Yes

Are all the source data underlying the results available to ensure full reproducibility?

Yes

Is the study design appropriate and is the work technically sound?

Yes

Are the conclusions drawn adequately supported by the results?

Partly

Are sufficient details of methods and analysis provided to allow replication by others?

Yes

Reviewer Expertise:

neurogenetics, ciliopathies

I confirm that I have read this submission and believe that I have an appropriate level of expertise to confirm that it is of an acceptable scientific standard.

# Peer review: Reviewer response for version 1

**DOI:** 10.5256/f1000research.43403.r81403

**Published:** 2021-04-06

**Authors:** Paraskevi Goggolidou

**Affiliations:** 1 Department of Biomedical Science and Physiology, Faculty of Science and Engineering, University of Wolverhampton, Wolverhampton, UK

This manuscript by Al Alawi *et al.* provides an interesting application of Whole Exome Sequencing (WES) in Ciliopathy patients in Jordan. The authors have looked at a small number of Ciliopathy patients and identified mutations of interest in 50% of them. As they are working with rare disease patients, the limitations in sequencing bigger patient populations are understandable. Furthermore, this work provides a very useful proof of concept in the application of WES for genetic diagnosis of rare diseases, such as Ciliopathies.

 However, as discussed in the discussion section, WES has got limitations and other approaches such as WGS might have been more insightful for this study. The manuscript would thus benefit from a more thorough discussion of the possibility of identified by WGS cases of non-exonic mutations causing Ciliopathies and a comment on the mechanisms behind this. Furthermore, it is important to comment on whether there were any single heterozygous pathogenic variants identified in known genes and if they checked for genes within stretches of homozygosity in consangeneous families. Finally, for the non-specialist audience a brief description of the key characteristics and genes associated with renal ciliopathies in the introduction section would have been useful.

Is the work clearly and accurately presented and does it cite the current literature?

Yes

If applicable, is the statistical analysis and its interpretation appropriate?

Not applicable

Are all the source data underlying the results available to ensure full reproducibility?

Yes

Is the study design appropriate and is the work technically sound?

Yes

Are the conclusions drawn adequately supported by the results?

Partly

Are sufficient details of methods and analysis provided to allow replication by others?

Yes

Reviewer Expertise:

Renal Ciliopathies

I confirm that I have read this submission and believe that I have an appropriate level of expertise to confirm that it is of an acceptable scientific standard, however I have significant reservations, as outlined above.

# Response

**Published:** 2021-06-30

**Authors:** John Sayer

**Affiliations:** Institute of Genetic Medicine, Newcastle University, UK

We agree that WGS may be more powerful than WES in order for us to solve the unsolved cases. We have added some discussion regarding this point as suggested. In response to the comments regarding the “negative cases’ we have now expanded the paper to discuss these further as there may, as the reviewer suggests be some useful learning points. As all the families were consanguineous, we focussed on homozygous variants but we have commented now on any significant heterozygous variants in cystogenes. Similarly, we have looked at missense alleles and synonymous changes that have been excluded by pathogenicity filters and comment on these also. Finally, as suggested we report homozygous variants in regions of homozygosity by descent in candidate genes that may shed new light on renal ciliopathies. A new table detailing variants in unsolved cases has been added and the discussion expanded to account for these new data.

 We have now added a brief introduction to renal ciliopathies and the common genetic causes has now been added as suggested.

# Peer review: Reviewer response for version 1

**DOI:** 10.5256/f1000research.43403.r81405

**Published:** 2021-03-30

**Authors:** Enza Maria Valente

**Affiliations:** 1 Neurogenetics Research Center, IRCCS Mondino Foundation, Pavia, Italy; 2 Department of Molecular Medicine, University of Pavia, Pavia, Italy

In this short article, the authors show the importance of Whole Exome Sequencing (WES) approach as a first genetic screening for patients with renal ciliopathy syndromes. They focus on a small cohort of 8 consanguineous Omani probands, who underwent WES. The diagnostic yield was 50%: four out of 8 probands were found to carry homozygous pathogenic variants in known genes. The study was carried out in a proper way, with sound methodology. The identified variants were adequately named and classified following ACMG guidelines. The discussion correctly addresses limitations of WES. 

 There are two main limitations in this article. The first is the very small cohort size, which does not really allow making a correct estimate of the diagnostic yield of WES in renal ciliopathies. Expansion of the study to a larger group of patients would clearly provide more useful information, also regarding the genetic background of genetic renal ciliopathies in Oman. Second, even maintaining this cohort, it would be very interesting to know more about “negative” cases: where there any single heterozygous pathogenic variants in known genes? Any missense variants classified as VUS? Moreover, since families were consanguineous, the authors should report whether they checked for genes included within stretches of homozygosity by descent, and list the potentially interesting homozygous variants in candidate genes lying within these regions. This would definitely improve the study which, in its present form, does not add substantial new data to current knowledge on the topic, and therefore it remains largely confirmatory.

Is the work clearly and accurately presented and does it cite the current literature?

Yes

If applicable, is the statistical analysis and its interpretation appropriate?

Yes

Are all the source data underlying the results available to ensure full reproducibility?

Yes

Is the study design appropriate and is the work technically sound?

Yes

Are the conclusions drawn adequately supported by the results?

Partly

Are sufficient details of methods and analysis provided to allow replication by others?

Yes

Reviewer Expertise:

neurogenetics, ciliopathies

I confirm that I have read this submission and believe that I have an appropriate level of expertise to confirm that it is of an acceptable scientific standard, however I have significant reservations, as outlined above.

# Response

**Published:** 2021-06-30

**Authors:** John Sayer

**Affiliations:** Institute of Genetic Medicine, Newcastle University, UK

Yes we agree that this is a small study size, but we were careful only to include subjects that had not had any previous genetic investigations and fulfilled the criteria of a suspected renal ciliopathy syndrome. In this way this study was seeing the added value in terms of genetic diagnosis to perform whole exome sequencing as a first line approach in contrast to a targeted renal genetics panel. We have added some comments regarding the size of the cohort.

 In response to the comments regarding the “negative cases’ we have now expanded the paper to discuss these further as there may, as the reviewer suggests be some useful learning points. As all the families were consanguineous, we focussed on homozygous variants but we have commented now on any significant heterozygous variants in cystogenes. Similarly, we have looked at missense alleles and synonymous changes that have been exclude by pathogenicity filters and comment on these also. Finally, as suggested we report homozygous variants in regions of homozygosity by descent in candidate genes that may shed new light on renal ciliopathies. A new table detailing variants in unsolved cases has been added and the discussion expanded to account for these new data.
